# A Novel, Molybdenum-Containing Methionine Sulfoxide Reductase Supports Survival of *Haemophilus influenzae* in an *In vivo* Model of Infection

**DOI:** 10.3389/fmicb.2016.01743

**Published:** 2016-11-14

**Authors:** Rabeb Dhouib, Dk. Seti Maimonah Pg Othman, Victor Lin, Xuanjie J. Lai, Hewa G. S. Wijesinghe, Ama-Tawiah Essilfie, Amanda Davis, Marufa Nasreen, Paul V. Bernhardt, Philip M. Hansbro, Alastair G. McEwan, Ulrike Kappler

**Affiliations:** ^1^Centre for Metals in Biology/Australian Infectious Diseases Research Centre, School of Chemistry and Molecular Biosciences, The University of Queensland, St. LuciaQLD, Australia; ^2^Centre for Asthma and Respiratory Diseases, Hunter Medical Research Institute, The University of Newcastle, New LambtonNSW, Australia; ^3^Department of Chemistry and Biochemistry, The University of Arizona, TucsonAZ, USA

**Keywords:** molybdenum enzymes, *Haemophilus influenzae*, methionine sulfoxide reductase, host–pathogen interaction, DMSO reductase enzyme family

## Abstract

*Haemophilus influenzae* is a host adapted human mucosal pathogen involved in a variety of acute and chronic respiratory tract infections, including chronic obstructive pulmonary disease and asthma, all of which rely on its ability to efficiently establish continuing interactions with the host. Here we report the characterization of a novel molybdenum enzyme, TorZ/MtsZ that supports interactions of *H. influenzae* with host cells during growth in oxygen-limited environments. Strains lacking TorZ/MtsZ showed a reduced ability to survive in contact with epithelial cells as shown by immunofluorescence microscopy and adherence/invasion assays. This included a reduction in the ability of the strain to invade human epithelial cells, a trait that could be linked to the persistence of *H. influenzae*. The observation that in a murine model of *H. influenzae* infection, strains lacking TorZ/MtsZ were almost undetectable after 72 h of infection, while ∼3.6 × 10^3^ CFU/mL of the wild type strain were measured under the same conditions is consistent with this view. To understand how TorZ/MtsZ mediates this effect we purified and characterized the enzyme, and were able to show that it is an S- and N-oxide reductase with a stereospecificity for S-sulfoxides. The enzyme converts two physiologically relevant sulfoxides, biotin sulfoxide and methionine sulfoxide (MetSO), with the kinetic parameters suggesting that MetSO is the natural substrate of this enzyme. TorZ/MtsZ was unable to repair sulfoxides in oxidized Calmodulin, suggesting that a role in cell metabolism/energy generation and not protein repair is the key function of this enzyme. Phylogenetic analyses showed that *H. influenzae* TorZ/MtsZ is only distantly related to the *Escherichia coli* TorZ TMAO reductase, but instead is a representative of a new, previously uncharacterized clade of molybdenum enzyme that is widely distributed within the Pasteurellaceae family of pathogenic bacteria. It is likely that MtsZ/TorZ has a similar role in supporting host/pathogen interactions in other members of the Pasteurellaceae, which includes both human and animal pathogens.

## Introduction

An emerging aspect of bacterial pathogenesis that is receiving increased attention is the role of metabolic interactions between the host and pathogen (Rhee et al., 2011; Bliska and Van Der Velden, 2012; Grubmüller et al., 2014; Heroven and Dersch, 2014; Hofreuter, 2014; Othman et al., 2014). We have previously analyzed the metabolic properties of *Haemophilus influenzae* (HI), a host adapted human pathogen of the family *Pasteurellaceae* that causes or contributes to a diverse array of upper and lower respiratory tract infections (Eldere et al., 2014; Othman et al., 2014). As is typical for a host-adapted pathogen, about 60–80% of healthy children and a high percentage of adults are asymptomatic carriers of HI (Mukundan et al., 2007). At the same time HI is one of the most common pathogens contributing to chronic and acute otitis media, as well as diseases of the lower respiratory tract such as chronic obstructive pulmonary disease (COPD), asthma and pneumonia (Wood et al., 2010; Essilfie et al., 2011, 2015; Tay et al., 2015).

Our previous analyses indicated that strain- as well as niche-specific factors including varying oxygen tension influence the metabolite profile of HI strains and also alter gene expression profiles, especially in genes involved in central carbon metabolism and the respiratory chain (Othman et al., 2014). These results suggested that the energy generation processes in HI provide the adaptability required for specific niches, e.g., during infection of the middle-ear or in biofilms, which would be mostly anaerobic, or for colonization of the more aerobic environment of the nasopharynx and respiratory tract, while specific, strain related adaptations may confer the ability to cause disease in these particular body niches.

A gene showing particularly striking changes in expression between the HI RDKW20 (HIRD) laboratory reference strain (Fleischmann et al., 1995) and the non-typeable COPD clinical isolate strain HI 2019 (Campagnari et al., 1987) (HI2019) was the *torZ* gene, that encodes a putative trimethylamine-N-oxide (TMAO) reductase. In both HIRD and HI2019 strains expression levels of *torZ* were maximal under anaerobic conditions but in HI2019 the observed levels of expression were significantly higher than in HIRD (Othman et al., 2014). A gene encoding a distantly related, S- and N-oxide reducing enzyme, *dmsA*, did not show increased expression in the HI2019 strain compared to HIRD (Othman et al., 2014). The *torZ*-encoded enzyme belongs to the DMSO reductase family of mononuclear molybdenum enzymes and these enzymes have been shown to have important roles in a variety of pathogenic bacteria. For example, a gene knockout in a tetrathionate reductase in *Salmonella enterica* serovar Typhimurium has been shown to attenuate virulence (Contreras et al., 1997; Bliska and Van Der Velden, 2012; Rivera-Chávez et al., 2013), while a strain of *Mycobacterium bovis* BCG carrying a gene knockout in a molybdenum-containing nitrate reductase showed reduced survival in immune-compromised mice (Fritz et al., 2002). Inactivation of the nitrate reductase in *M. tuberculosis* led to increased sensitivity to acid and nitrogen stress and reduced survival when respiration was inhibited (Sohaskey, 2008; Tan et al., 2010). In *Campylobacter jejuni* expression of a Mo-containing formate dehydrogenase was shown to be essential for successful invasion and adherence of the bacteria to Caco-1 cells (Pryjma et al., 2012). We therefore hypothesized that the increased expression levels of the *torZ* gene in the clinical isolate strain HI2019 could be indicative of a role of TorZ in HI pathogenesis.

When compared to known *Escherichia coli* Mo-containing S- and N-oxide reductases, the protein encoded by the HI *torZ* gene shows the highest sequence identity (58%) to the TorZ/BisZ protein that was initially described as a second, minor biotin sulfoxide reductase (BisZ) based on amino acid sequence similarities (Del Campillo Campbell and Campbell, 1996). A later study found that the *E. coli* enzyme was located in the periplasm, not the cytoplasm, and formed an operon with a gene encoding a pentaheme cytochrome, *torY*, related to the TorC cytochrome of the main *E. coli* TMAO respiratory system encoded by the *torCAD* operon (Gon et al., 2000). While an *E. coli* strain carrying only the *torYZ* operon was unable to grow by TMAO respiration, overexpression of *torYZ* in the same strain enabled anaerobic growth in the presence of TMAO, dimethyl sulfoxide (DMSO) and biotin sulfoxide (BSO) (Gon et al., 2000). As anaerobic growth with TMAO led to the highest cell densities, it was concluded that TorYZ was a TMAO respiratory system related to TorCA (Gon et al., 2000). However, two important differences between the TorZ and the TorA TMAO reductases were noted. TorA requires a specific chaperone, TorD, for maturation which appears not to be required for the formation of TorZ, and secondly, TorZ is able to reduce S- and N-oxides, while TorA will only convert N-oxides (Gon et al., 2000). The classification of these enzymes as true N-oxide reductases or combined S- and N-oxide reductases is based on the presence of crucial active site residues, a tyrosine and a tryptophan. True N-oxide reductases like TorA lack the tyrosine residue and are unable to reduce S-oxides, while all combined S- and N-oxide reductases contain both active site residues (Ridge et al., 2004; Cobb et al., 2007). No further work on the *E. coli* TorZ protein was reported, and the physiological role of TorYZ in *E. coli* is still unknown.

Here we report the characterization of the HITorZ enzyme and its effects on virulence and host–cell interactions. We were able to demonstrate that TorZ from HI is a novel type of molybdenum enzyme that converts methionine sulfoxide (MetSO) as its preferred substrate, but appears to be unable to repair MetSO damage in proteins. A *torZ* gene knockout showed reduced biofilm formation and in biofilm survival *in vitro*, as well as impaired host cell interactions and reduced survival in a murine model of infection, clearly indicating a role for TorZ in host–pathogen interactions.

## Materials and Methods

### Growth of Bacterial Strains

*HI* 2019 wild-type (HI2019^WT^) (Campagnari et al., 1987) and derivatives of this strain were cultivated on supplemented brain heart infusion (sBHI) agar (Becton Dickinson) that contained 10 μg/mL hemin (Johnston, 2010) and 10 μg/mL β-NAD at 37°C with 5% CO_2_. A chemically defined growth medium (sRPMI) was also used and contained 25 mM HEPES, pH 7.3, 0.8 mM sodium pyruvate, 0.08 mg/mL uracil, 0.17 mg/mL inosine, 8 μg/mL β-NAD, 17 μg/mL hemin, and 2 mg/mL NaHCO_3_ in RPMI1640 (Sigma–Aldrich) (Coleman et al., 2003). *E. coli* DH5α (Life Technologies) was grown in Luria Bertani (LB) broth or on LB agar (Sambrook et al., 1989) at 37°C. Kanamycin (kan) (100 μg/mL *E. coli*; 10 μg/mL HI), spectinomycin (spec) (50 μg/mL *E. coli*; 20 μg/mL HI), and ampicillin (100 μg/mL *E. coli*) were added to culture media when required. Growth experiments used sRPMI medium and aerobic, microaerophilic and anaerobic growth conditions (Cooper et al., 2003; Othman et al., 2014) as described in (Othman et al., 2014; Dhouib et al., 2015). Growth data were collected every 1–2 h up to 12 h with a final reading at 24 h. Growth rates were determined using an online tool ^1^.

### Molecular and Biochemical Methods

Standard methods were used throughout (Ausubel et al., 2005), all chemicals were purchased in analytical grade unless otherwise indicated. Plasmid and PCR product purification used the Purelink (l249) Plasmid DNA miniprep Kit (Life Technologies) and the Purelink PCR purification kit (Life Technologies). Restriction Enzymes were from Life Technologies, T4 Ligase and RNAse inhibitor were from Promega. Competent *E. coli* cells were prepared as described previously (Hanahan et al., 1991). Genomic DNA was isolated using the Genomic DNA mini kit (Life Technologies). General PCR used GoTaq Master Mix Green (Promega), high fidelity amplifications used Phusion master Mix (Finnzymes). RNA isolation used the Illustra RNAspin mini kit (GE Healthcare, Biosciences) and RNAprotect bacteria reagent (Qiagen), the Turbo-DNA-free kit (Life Technologies) and Superscript III (Life Technologies) to prepare cDNA as described previously (Othman et al., 2014). SDS-PAGE gels were prepared according to the method of Laemmli (1970). Protein concentrations were determined using the BCA-1 kit (Sigma–Aldrich). Small-scale cell extracts of *E. coli* and *HI* were prepared using BugBuster Master Mix (Novagen) as per the manufacturer’s instructions. Protein concentrators (Vivaspin MWCO 50 kDa) were from GE Healthcare Biosciences.

Periplasmic fractionation used the osmotic shock method essentially as described in Rodden and Scocca (1972). Following removal of the periplasmic protein fraction spheroplasts were lysed using BugBuster Master Mix (Novagen) to generate the cytoplasmic/membrane protein fraction. Analysis of molybdenum contents of purified HIrTorZ was carried out at Analytical Services of the School of Agriculture and Food sciences at the University of Queensland using ICP-OES.

#### Enzyme Assays

Sulfoxide activities in crude extracts from anaerobically grown cells or using purified proteins were assayed at 37°C by monitoring the oxidation of reduced benzyl viologen radical monocation at 600 nm (𝜀_600_ = 7.4 mM^-1^ cm^-1^) (Dickie and Weiner, 1979). Enzyme assays were performed anaerobically in 20 mM sodium phosphate buffer (pH 6.8) containing 0.2 mM benzyl viologen, 0.3 mM sodium dithionite and one of the following terminal electron acceptors: 17 mM DMSO, 10 mM racemic DL-methionine sulfoxide (racemic MetSO or DL- MetSO), 10 mM L-MetSO, 5 mM of two S-based diastereomers, S(*R*)-*S*-biotin sulfoxide and S(*S*)-*S*-biotin sulfoxide (*R*-BSO and *S*-BSO), and 20 mM TMAO. For kinetic assays the concentrations of the substrates were varied. The diastereomers S(*R*)-*S*-biotin sulfoxide and S(*S*)-*S*-biotin sulfoxide were prepared as described in Melville (1954) and Melville et al. (1954) and their isomeric purity (>95%) was confirmed by ^1^H NMR.

Specific enzyme activities are given as μmoles of substrate reduced per min (U) and mg of protein present, kinetic data were fitted using either Sigma Plot 12 (Systat) or Prism 6.0 (GraphPad).

#### Construction and Complementation of a HI2019^Δ^*^torZ^* Strain

Two DNA fragments (∼1200 bp each) covering the *torZ* gene were amplified from HI2019 genomic DNA and primer pairs (i) HI_torZ_extF and HI_torZ_intR and (ii) HI_torZ_extR and HI_torZ_intF (**Table 1**). The obtained fragments were digested with *Bam*HI before being used in a three way ligation with pGEMT^®^-easy (Promega) to create pGEM-*torZ*. The kanamycin (kan) resistance cassette was amplified from the pUC-4K plasmid (Vieira and Messing, 1982) using primers Kan-BamHI-F and Kan-BamHI-R (**Table 1**) followed by insertion into the pGEM-*torZ Bam*HI site yielding pGEM-*torZ::Kan*. The resulting plasmid was linearized using *Sca*I and transformed into competent HI2019 using the method described in Poje and Redfield (2003) generating HI2019^Δ^*^torZ^* (genotype: HI2019 Kan^r^ *torZ::kan)* following selection on sBHI 20 μg/mL kan agar plates. The inactivation of *torZ* was confirmed by PCR.

**Table 1:** Sequences of oligonucleotide primers used in this study.

Primer name	Sequence
Kan-BamHI-F	5′ –AAAA GGA TCC GGA AAG CCA CGT TGT GTC –3′
Kan-BamHI-R	5′ –AAAA GGA TCC CTG AGG TCT GCC TCG TGA –3′
HI_torZ_extF	5′ –TTACGCCACCTGTTTAGG –3′
HI_torZ_extR	5′ –ATGAAAAAGAATAACGTAAA –3′
HI_torZ_intF	5′ –AAAAGGATCCCGCTTTGCCTGATGGACT –3′
HI_torZ_intR	5′ –AAAAGGATCCGGGGCAAAAACATGGTTG –3′
HI2019torZcomp_Xma_F	5′–AAAACCCGGGGGACATTACAAACCGGCAGA –3′
HI2019torZcomp_Xma_R	5′–AAAACCCGGGCCATCTCACCACAATCAGTGTG –3′
HIbisC-SP_pPro_Bam_F	5′ –AAAAGGATCCAAAGAAGCTGAAATGAAAACG –3′
HIbisC_pPro_Xba_R	5′ –AAAATCTAGATTACGCCACCTGTTTAGG –3′
HItorZ_torY R	5′ —CCTTTATGCCCCACAAAACCACC —3′
HItorZ_torY F	5′ –GTTCAAGGCACCTTGCATGGTTG –3′
HI2019_Qp_gyrAF	5′ –GCGTGCATTACCTGACGTTCGAG –3′
HI2019_Qp_gyrAR	5′ –CCCACAACACGCGCTGATTTTAC –3′

To complement the HI2019^Δ^*^torZ^* mutant, the *torYZ* operon (4450 bp) was amplified using primers HI2019torZcomp_Xma_F and HI2019torZcomp_Xma_R (**Table 1**) and cloned into p601.1-Sp2 (Johnston et al., 2007) using the *Xma*I site. The plasmid was linearized using *Bam*HI and transformed as described above, and HI2019^Δ^*^torZ^*^_comp^ selected on sBHI plates containing 20 μg/mL kanamycin and 50 μg/mL spectinomycin. Correct integration of the construct was confirmed by PCR.

#### Construction of a TorZ Overexpression Plasmid

A pProexHtb (Invitrogen) based protein expression plasmid containing the *torZ* gene without the region coding for the signal peptide was constructed using primers HIbisC-SP_pPro_Bam_F and HIbisC_pPro_Xba_R (**Table 1**) and the restriction enzyme sites introduced during high fidelity PCR. The resulting ligation products were transformed into DH5α (Life Technologies) to obtain pProex HI*torZ*-SP, which was verified by DNA sequencing.

Optimal expression of the recombinant TorZ (rTorZ) protein was obtained using pProex HI*torZ*-SP in *E. coli* DH5α and LB medium supplemented with 1 mM sodium molybdate and 100 μg/mL ampicillin. Overexpression cultures (100 mL supplemented LB in a 250 mL shake flask) were inoculated from overnight cultures to an OD_600_ of 0.05–0.1, followed by incubation with shaking at 37°C, with shaking at 200 rpm until an OD_600_ of 0.6–0.8 was reached. IPTG was added to a final concentration of 100 μM and the cultures incubated at 30°C, 200 rpm overnight before harvesting of cells by centrifugation (3000 × *g*, 4°C, 10 min). Cell pellets were stored at -20°C.

#### Purification of Recombinant TorZ

Recombinant TorZ (rTorZ) was purified from 4 L of protein expression culture. The cell pellets were resuspended in 20 mM Tris-Cl pH 8.0, 5% glycerol, 1 mM Pefabloc SC (Roche) (∼5 mL/g wet weight) and lysed by three passes through a French Press (Aminco) at 15000 psi. Imidazole was added to a concentration of 20 mM, followed by removal of cell debris by centrifugation at 30 000 × *g*, 4°C, 30 min. The resulting cell-free extract and was loaded onto a 5 mL HiTrap Ni-NTA Sepharose column (GE Healthcare Biosciences) equilibrated with 20 mM K-Phosphate pH 7.4, 500 mM NaCl, 20 mM imidazole (buffer A). The elution buffer (buffer B) was the same as buffer A but contained 500 mM imidazole. Following loading of the sample, the column was washed with 5 CV of buffer A, followed by a linear gradient (0–100% buffer B) over 10 and 2 CV of 100% buffer B. SDS-PAGE was used to assess the purity of fractions, followed by pooling according to rTorZ purity. Protein pools were concentrated and separated on a Superdex 200 HiLoad (16/60) gel filtration column (GE Healthcare Biosciences) equilibrated in 20 mM Tris-Cl pH 7.8, 150 mM NaCl, 5% glycerol. Separated proteins were again pooled according to purity, concentrated and desalted using a PD-10 column (GE Healthcare Biosciences) equilibrated in 20 mM Tris-Cl pH 7.8 5% glycerol. Purified proteins were stored at -80°C until further use.

#### Purification of *E. coli* MsrP for Calmodulin Assays

The *E. coli* YedY protein has recently been proposed to be renamed to MsrP based on its ability to repair MetSO damage in periplasmic proteins, the two names refer to the same protein (Gennaris et al., 2015). *E. coli* strain JM109 λpir harboring the plasmid pMSYZ3 for expression of His_6_-Tagged MsrP and native YedZ was kindly provided by Prof. Joel Weiner (University of Alberta, Edmonton, AB, Canada). *E. coli* MsrP-6xHis was purified as described in Loschi et al. (2004).

#### *In vitro* Repair of Oxidized Calmodulin (CaMox)

Calmodulin (CaM) (Astralscientific, Cat.N. 05-0103-2) was decalcified by dissolving in 50 mM Hepes, pH 7.5, 10 mM EDTA (CaM conc.: 150 μM), followed by treatment with 50 mM H_2_O_2_ for 4 h at room temperature. H_2_O_2_ was removed by gel filtration through PD MiniTrap G-10 (GE Healthcare) (Grimaud et al., 2001; Tsvetkov et al., 2005). CaM repair assays used 4 μM of purified enzyme (rTorZ or MsrP) in 250 μL containing oxidized CaM, 4 μM, 10 mM benzyl viologen and an excess of sodium dithionite (2 mM). Samples were incubated anaerobically at 37°C for an hour. Control reactions used both proteins, but without benzylviologen and dithionite. Sodium dithionite was removed from samples by four consecutive dilutions with 50 mM Hepes, pH 7.5 and concentration using Amicon^®^ Ultra-3K (Millipore) followed by SDS-PAGE (17.5%) analysis.

#### Quantitative RT-PCR (qRT-PCR) and *torYZ* Cotranscription Analysis

Quantitative RT-PCR (qRT-PCR) was performed essentially as described in (Othman et al., 2014) with three biological replicates for each culture condition. RNA was isolated from 2 mL of HI cultures (anaerobic: OD_600nm_ = 0.4, aerobic and microaerophilic: OD_600nm_ = 0.8) preserved in RNAprotect bacteria (Qiagen). gDNA was removed using the Turbo DNA-free^TM^ Kit (Life Technologies), cDNA was synthesized using 500 ng of RNA for the combined biological replicate samples using Superscript III (Life Technologies) and random hexamer primers (Life Technologies). RNA concentrations were determined using the Quant-IT RNA kit (Life Technologies). For RNA isolation from co-cultures planktonic cells were preserved directly in RNAprotect. RNA from adherent and internalized HI was preserved in RNA protect following harvesting and water lysis of the epithelial cells with subsequent collection of the bacteria by centrifugation. qRT-PCR reactions (10 μL) used diluted cDNA (1:100–1:1000) as template, SYBR green Master Mix (Applied Biosystems) and primers described previously (Othman et al., 2014). The *gyrA* gene was used as the reference gene and data analysis and normalization was performed as in (Kappler et al., 2005). Co-transcription of the *torYZ* genes was tested using cDNA derived from anaerobically grown cultures using primers HI*torY-torZ* F and HI*torY-torZ* R (**Table 1**) and GoTaq Master Mix green (Promega). Genomic DNA was used as the positive control.

### Biofilm and Biomass Quantification Assays

*Haemophilus influenzae* (anaerobic, microaerophilic, and aerobic condition) were grown to an OD_600nm_ of 0.2–0.3in sBHI at 37°C. Cultures were diluted to an OD_600nm_ of 0.05, before being distributed into 96-well microtiter plates (125 μL per well) (U-bottom, polystyrene, TechnoPlas) and incubation (24 h, 37°C) with or without shaking. Anaerobic jars were used for anaerobic incubations. For biofilm detection, planktonic cells were removed by careful washing with water, bound cells detected using crystal violet as described in Schembri and Klemm (2001). For biomass quantification, planktonic cells were removed by washing with sterile water, and the bound cells were incubated for 10 min with 200 μL 0.1 mg/mL proteinase K in 1x PBS (Izano et al., 2009). The detached bacteria were mixed thoroughly by vigorous pipetting, serially diluted in 1x PBS and plated on sBHI agar to estimate the number of colony-forming units (CFU) per well. Statistical comparisons of mean CFU/well and absorbances were performed by one-way ANOVA using the Tukey *post hoc* method (Prism 7 software package, GraphPad). Additional analyses were carried out using two tailed *t*-tests. A *p* < 0.05 was considered statistically significant.

### HOCl Susceptibility Assay

The assay was carried out essentially as described by Benoit et al. (2013). HI2019^WT^ and derivative strains were grown overnight on BHI agar plates, the cell material was harvested using an inoculation loop and resuspended in 1X PBS to an OD_600_ of 1.1. To 1.8 ml cell suspension in a 10 ml tube, 0.2 ml of water or freshly prepared 10x HOCl stock solutions were added (final OD = 1.0 in sample), and samples incubated at room temperature with shaking for 60 min prior to serial dilution and determination of CFU/ml as described above. Final HOCl concentrations in samples ranged from 0.05 to 0.5 mM. Statistical comparisons of mean CFU/ml were performed by two-way ANOVA (Prism 7 software package, GraphPad). Additional analyses were carried out using two tailed *t*-tests. A *p* < 0.05 was considered statistically significant.

### Tissue Culture, Adherence and Invasion Assays and Neutrophil Killing Assays

Human bronchial epithelial 16HBE14 cells (Gruenert et al., 1988), kindly provided by Dr. Kirsten Spann (Queensland University of Technology), were propagated in MEM – GlutaMAX^TM^ (Gibco^®^, Life Technologies), supplemented with 10% fetal calf serum (Gibco^®^, Life Technologies, Cat.-No. 16000-044) (sMEM). The cells were seeded into individual wells of 24-well culture dishes (Greiner Bio-One, Cat.-No. 662160) at an approximate density of 2^∗^10^5^ cells/well, incubated at 37°C with 5% CO_2_ until reaching confluence, then used for infection studies. Bacterial adherence and invasion was determined using a standard gentamicin-survival assay (St Geme and Falkow, 1990), as described previously (Dhouib et al., 2015). Fresh overnight cultures of HI2019^WT^, HI2019^ΔmobA^ and HI2019^ΔmobA_comp^ were prepared on sBHI-agar plates. The bacteria were resuspended in 5 mL sMEM and then diluted in the same culture medium to 2^∗^10^7^ bacteria/mL. Confluent 16HBE14 monolayers in 24-well culture dishes were washed once with fresh pre-warmed sMEM and infected using a multiplicity of infection (MOI) of 1:100 (epithelial cells: HI). The infected monolayers were incubated for 4 or 24 h at 37°C with 5% CO_2_, washed three times with prewarmed sMEM before sMEM containing gentamicin (50 μg/mL) was added followed by incubation for 1 h at 37°C, 5% CO_2_. The monolayers were washed three times with fresh sMEM and lysed by the addition of sterile 1% (w/v) saponin in 1x PBS (pH 7.4). The epithelial cell lysates were mixed thoroughly by vigorous pipetting and serially diluted in BHI broth. Dilutions (5 μL of 10^0^–10^-6^ dilutions) were plated on sBHI agar and incubated overnight to estimate the numbers of colony-forming units (CFU) per well. Experiments determining bacterial adherence and invasion were carried out in the same way but omitting the gentamicin incubation.

Neutrophil killing assays were also carried out as in Dhouib et al. (2015). Human neutrophils were isolated and purified from venous blood using the PolyMorphPrep kit (Axis-Shield) as per the manufacturer’s instructions and seeded into 96-well plates at 2^∗^10^5^ cells/well. HI strains grown overnight on fresh sBHI-agar plates were resuspended in RPMI medium containing 2% heat inactivated autologous human plasma, diluted to 2^∗^10^7^ CFU/mL in the same medium and then added to neutrophils at an MOI of 1:10 (neutrophils: HI) (Walker et al., 2007). Plates were centrifuged at 500 × *g* for 10 min then incubated at 37°C with 5% CO_2_ for 2 h. After incubation, neutrophils were lysed with water, the content of each well serially diluted in BHI and plated on sBHI-agar for overnight incubation and enumeration of CFU. *E. coli* DH5α (Life Technologies) was used as a positive control. Percent survival of bacteria was calculated as ([CFU/ml experimental well]/[CFU/ml initial inoculum])^∗^100. Statistical analyses were carried out with Prism7 (GraphPad) using One Way ANOVA (α = 0.05) and SIDAK’s multiple comparison test.

### Immunofluorescence Staining

16HBE14 cells were grown to confluence on glass coverslips (13 mm, Number#1, ProSciTech), placed in 24-well plates (Greiner Bio-One, Cat.-No. 662160) and then infected with HI strains as described for adherence and invasion assays. After 4 or 24 h of incubation at 37°C with 5% CO_2_, planktonic cells were removed by washing three times with 1x PBS. Epithelial cells and bacteria were then fixed in 4% paraformaldehyde for 15 min, permeabilized with 0.1% Triton X-100 in 1x PBS for 5 min, and blocked overnight at 4°C with blocking buffer (2% BSA, 0.02% sodium azide in 1x PBS). Immunofluorescence staining of HI was performed using the primary antibody 6E4 (200 μL of 1:100 dilution) kindly provided by Prof. Michael Jennings (Institute for Glycomics, Griffith University, Australia) (Erwin et al., 2006). After 3 h of incubation at room temperature, the wells were washed three times with 500 μL of blocking buffer before addition of 200 μL of a 1:100 dilution of the secondary antibody Anti-mouse IgG (whole molecule)-FITC antibody produced in goat (Sigma–Aldrich) and incubation for 2 h in the dark. Epithelial cells were stained with CellTracker^TM^ Orange CMTMR fluorescent dye (Life Technologies) (200 μL of 1 μg/mL solution) for 1 h at room temperature in the dark, coverslips were mounted onto slides using ProLong^®^ Gold antifade reagent (Life Technologies) and images were acquired using an Axiophot 2 epifluorescence light microscope (Zeiss).

### Murine Infection Assays

Experimental animal procedures were carried out in strict accordance with the recommendations in the NSW Animal Research Regulation 2005, and the Australian code of practice for the care and use of animals for scientific purposes of the National Health. Protocols were approved by the Animal Care and Ethics Committees of the University of Newcastle and the University of Queensland. For HI pulmonary infection, a mouse model described previously by Morey et al. (2013) was used. HI strains were grown in sBHI for 16 h at 37°C with 5% CO_2_. BALB/c female mice (5–6 weeks old) were inoculated intranasally with 30 μL of a bacterial suspension containing 10^7^ CFUs. Groups of 6 mice were euthanized and necropsied at 0, 24, 48, and 72 h.To quantify the bacterial recovery, lungs were aseptically removed, homogenized in 1 mL 1x PBS and serially diluted in the same buffer. Each dilution was plated onto sBHI plate incubated overnight at 37°C with 5% CO_2_ and CFUs per lung were calculated (Essilfie et al., 2011, 2012, 2015). Statistical comparisons of mean CFU/lung were performed by one-way ANOVA using the Tukey *post hoc* method as integrated into the Prism 6 software package. Additional analyses were carried out using two tailed *t*-tests. A *p* < 0.05 was considered statistically significant.

### Phylogenetic Analysis of TorZ-Related Protein Sequences

One thousand and five amino acid sequences of proteins related to Molybdenum containing S- and N-oxide reductases from (see Supplementary Data) were retrieved from GenBank using BLASTP searches with different sulfoxide reductases as the search models. To maximize coverage of this group of proteins, initially several data sets were compiled using *E. coli* TorA (acc. no. P33225), *Rhodobacter capsulatus* DorA (acc. no. 1E61), *E. coli* BisC (acc. no. P20099), *E. coli* TorZ (acc. no. P46923) and *HI* TorZ (acc. no. P44798) as input sequences for database searches. Criteria for similarity were a query coverage or 80% or better combined with low *e*-values for the respective search models. Sequence lists were then combined and duplicates removed. Sequences were aligned using ClustalW as incorporated into the MEGA6.0 software package (Tamura et al., 2011), phylogenetic analyses (Neighbor joining, UPGMA, Minimum Evolution) and bootstrapping (500 cycles) were also carried out in MEGA 6.0.

### Ethics Statement

Experimental animal procedures were carried out in strict accordance with the recommendations in the NSW Animal Research Regulation 2005, and the Australian code of practice for the care and use of animals for scientific purposes of the National Health. Protocols were approved by the Animal Care and Ethics Committees of the University of Newcastle (A-2012-211) and the University of Queensland (UN/SCMB/335/13/NHMRC). Human blood for isolation of human primary neutrophils was specifically obtained for this study from healthy donor. All donors provided informed written consent. The procedure was approved by the University of Queensland Medical Research Ethics Committee (project number 2010000491).

## Results

### The *torYZ* Operon Is Conserved in *H. influenzae* Strains and Is Expressed under Oxygen-Limiting Growth Conditions

*Haemophilus influenzae* is known for its high genetic variability, however, our analyses showed that the *torYZ* operon is conserved in 65/80 (81%) of the available complete and partial genomes of HI strains, although in some cases one of the two genes was annotated as a pseudogene (Supplementary Table S1), possibly due to sequencing errors. The *torY* gene encodes a 366 amino acid (aa), membrane-bound *c*-type cytochrome with five heme groups related to the *E. coli* TorC (TIGR02162) cytochrome that is the electron donor to the TorA TMAO reductase (Ansaldi et al., 1999; Gon et al., 2001). TorC is a member of the NapC/NirT tetraheme cytochromes (pfam03264) that donate electrons to soluble nitrate and nitrite reductases, and a misinterpretation of NapC/NirT function is likely the reason why HI*torY* is erroneously annotated as encoding a ‘nitrate reductase’ in several of the available genomes.

**FIGURE 1:**
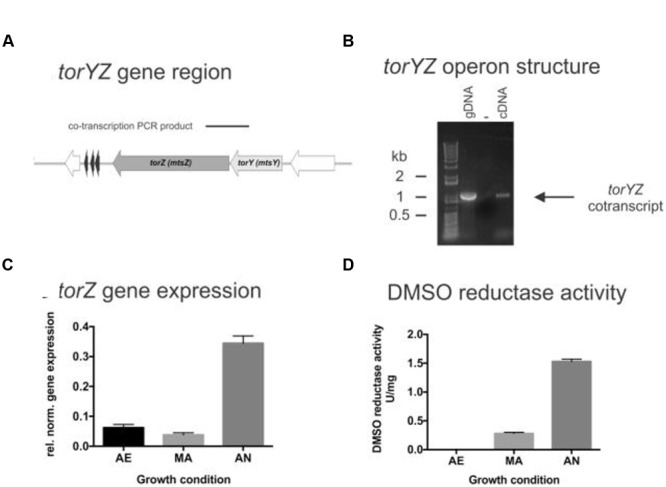
**Structure and function of the HI2019 *torYZ* operon. (A)** Schematic representation of the HI2019 *torYZ* gene region. The black bar indicates the position of the PCR product shown in **(B)**. **(B)** PCR based co-transcription analysis of HI2019 *torYZ*. ‘+’ = positive control containing gDNA; ‘-’ = no template control, C = cDNA (derived from anaerobic culture) as template. **(C)** Relative expression of the *torZ* gene in HI2019 cultures grown under aerobic (AE), microaerophilic (MA) and anaerobic (AN) growth conditions. Data were normalized using expression of the *gyrA* gene. **(D)** DMSO reductase activity in HI2019^WT^ Cell-free extracts derived from cultures grown under aerobic (AE), microaerophilic (MA), and anaerobic (AN) growth conditions. DMSO reductase activity in cell-free extracts is a relative measure of the activity of Mo-containing S-oxide reductases under the conditions tested. Errors are given as standard deviations of the mean, at least three repeat assay were carried out per condition.

The *torY* gene is separated by 25 bp from *torZ*, which encodes the molybdenum-containing catalytic subunit of the TorYZ system (**Figure 1**). TorZ is an 825 aa protein that belongs to the DMSO reductase family of molybdenum enzymes. The reaction catalyzed by TorZ is linked to the cellular Q-pool and thus the HI respiratory chain via its interactions with TorY, that uses reduced quinones as electron donors for subsequent reduction of TorZ. TorZ activity can thus influence both the cellular redox balance and energy generation, in addition to potential beneficial effects derived from the substrate converted by TorZ (**Figure 1**).

As expected, the HI2019 *torY* and *torZ* genes form an operon, as shown by a PCR-based co-transcription assay (**Figure 1**), and in HI2019 the operon is followed by hairpin loop (-5.3 kcal/mol, TATTA**TT**A**AAAAAG***AACGCA***CTTTTT**G**AA**GTGCG) that could serve as a potential transcription terminator. HI genomes generally also encode another Mo-containing sulfoxide reductase, the DmsABC protein that can catalyze reductions of similar compounds and has a catalytic subunit (DmsA) which has ∼30% sequence identity to TorZ.

To investigate the physiological function of TorZ, we assessed *torZ* gene expression and the presence of sulfoxide reductase activity in HI2019 crude extracts. Both assays clearly suggest a role for TorZ during anaerobic growth of both HI2019 and HIRD, with increased gene expression and DMSO reductase activity with decreasing levels of oxygen (**Figure 1**). However, it should be noted that the enzyme activities from crude extracts (**Figure 1**) reflect the combined activities of TorZ and DmsABC, as both enzymes react with the same enzyme assay chemistry, and cannot be distinguished in a crude extract. Moreover, it is interesting to note that neither of the main substrates described for TorZ and DmsA, i.e., TMAO and DMSO, respectively, are likely to be present in the human respiratory tract at high concentrations, raising the question of what the physiological substrate for HITorZ is. Possible candidates substrates are MetSO or BSO, and these were therefore included in activity tests.

### A HI2019^Δ^*^torZ^* Knockout Strain Is Not Susceptible to HOCl Induced Oxidative Stress But Shows Reduced Survival in Biofilms

**FIGURE 2:**
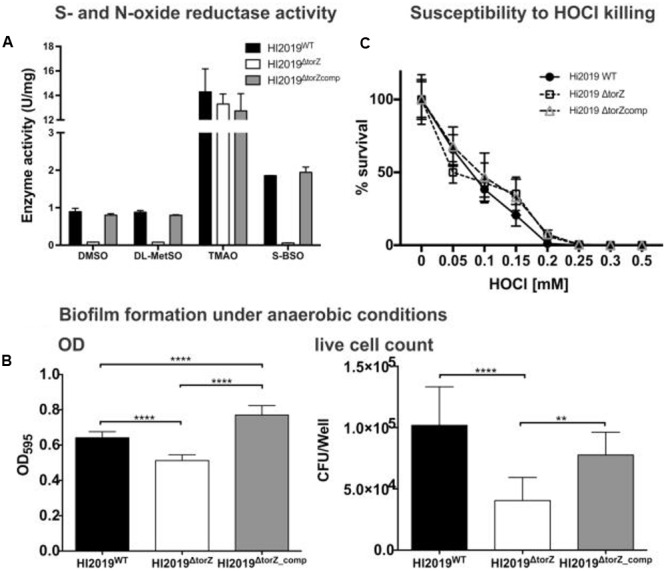
**Effects of the *torZ* gene inactivation on the physiology of HI2019^WT^, HI2019^Δ^*^torZ^* and HI2019^Δ^*^torZ^*^_comp^ strains. (A)** S- and N-oxide reductase activity in cell-free extracts of HI2019^WT^, HI2019^Δ^*^torZ^* and HI2019^Δ^*^torZ^*^_comp^ strains. Cell extracts were prepared from cultures grown anaerobically to ensure maximal expression of *torZ*, enzyme assays were carried out anaerobically using sodium dithionite reduced methylviologen as the electron donor to the enzymes and different S- and N-oxides. Each assay was repeated at least three times, experimental errors are given as standard deviations of the mean. **(B)** Biofilm formation and in biofilm survival of HI2019^WT^, HI2019^Δ^*^torZ^* and HI2019^Δ^*^torZ^*^_comp^ strains under anaerobic conditions. Left: Crystal violet based detection of biofilm density, Right: survival of HI in anaerobic biofilms measured as CFU present per well. Bacteria were recovered from biofilms using proteinase K treatment followed by serial dilution and plating. The data shown is one of three independent experiments carried out on different days, each bar represents the combined data of three biological replicates analyzed that day with eight replicate assays/biological replicate. One Way ANOVA was used to determine statistical significance. *P*-values: for all instances of ^∗∗∗∗^*p* < 0.0001, ^∗∗^*p* = 0.0077 (right panel, comparison *torZ* mutant and complemented strain). **(C)** Susceptibility of HI2019^WT^, HI2019^ΔtorZ^ and HI2019^ΔtorZ_comp^ strains to HOCl–induced oxidative stress. Bacteria were resuspended in 1xPBS (OD_600_ in assay = 1.0) and exposed to different concentrations of HOCl for 60 min before serial dilution and plating. The graph shows the combined data for three experiments carried out on three different days using a total of seven biological replicates for each strains. Error bars represent 95% confidence intervals, no significant differences in strain survival were detected (2-Way ANOVA).

A HI2019^Δ^*^torZ^* mutant was constructed to enable analyses of the physiological roles of TorZ. As expected, we clearly observed a loss of sulfoxide converting enzyme activities in HI2019^Δ^*^torZ^* (**Figure 2A**), with both DMSO and MetSO reductase activities reduced by ∼91%, while biotin sulfoxide reductase activity was reduced by 96%. TMAO reductase activity was not affected, indicating either that TorZ does not reduce TMAO or that a second enzyme system is present in the crude extracts that can carry out this reaction. The enzyme activities were restored to wild-type levels in a strain complemented for the *torZ* mutation, HI2019^Δ^*^torZ^*^_comp^ (**Figure 2A**). These data indicate that HI TorZ is the major S-oxide reductase in HI2019, and can convert a variety of substrates including DMSO and the two physiologically relevant compounds MetSO and biotin sulfoxide.

Despite the loss of the S-oxide reductase activity, on sRPMI- based medium supplemented with glucose the HI2019^Δ^*^torZ^* strain showed no growth defect, with growth rates showing no statistically significant different to the wild type (Supplementary Figure S1). In contrast, *in vitro* biofilm formation experiments using microtiter plates showed a reduced ability of HI2019^Δ^*^torZ^* to form biofilms under all conditions tested, and analysis of the CFUs present in the biofilm also revealed reduced survival of HI2019^Δ^*^torZ^* in the biofilm (**Figure 2B**; Supplementary Figure S2). As TorZ is a periplasmic enzyme and appears to be able to repair oxidatively damaged methionine that can form during infection/interaction of HI with host cells, we also tested the susceptibility of the strain to hypochlorite that can form easily in the presence of ROS, however, survival of HI2019^Δ^*^torZ^* in the presence of HOCl was the same as for the wild type (**Figure 2C**).

### Mutations in *torZ* Lead to a Reduced Ability of *H. influenzae* to Interact with Host Cells

Analyses of gene expression for HI2019^WT^ in co-culture with 16HBE14 epithelial cells revealed significant levels of *torZ* expression both in bacteria present in the tissue culture medium (‘planktonic’) and those adherent to the 16HBE14 cells (Supplementary Figure S3). Together with the observed biofilm formation defect this led us to hypothesize that HITorZ might affect interactions of the bacteria with host cells.

**FIGURE 3:**
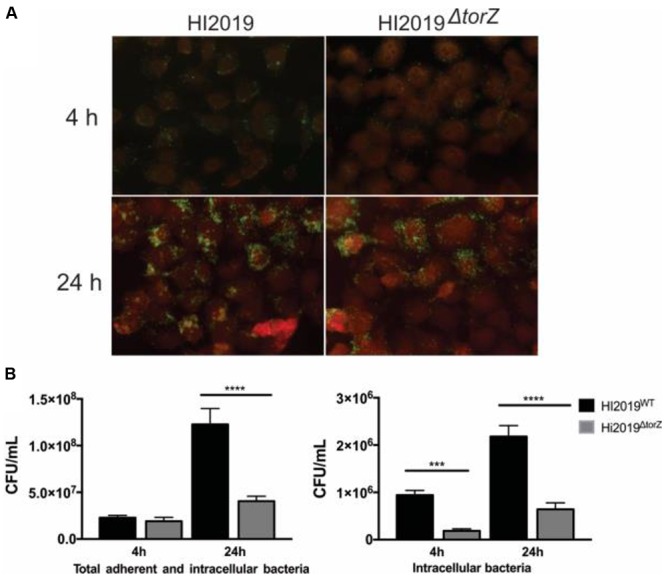
**Deletion of the TorZ protein leads to a change in the ability of HI2019 to colonize and invade human bronchial epithelial 16HBE14 cells. (A)** Immunofluorescence imaging of co-cultures of HI2019^WT^ and HI2019^Δ^*^torZ^*. Epithelial cells were stained using CellTracker^TM^, HI cells were detected using the antibody 6E4 (Erwin et al., 2006) as set out in the method section. **(B)** Total adherent and intracellular HI2019 cells present in co-cultures of 16HBE14 cells with HI2019^WT^ or HI2019^Δ^*^torZ^* after 4 and 24 h incubation. Cell numbers were determined by serial dilution plating and are reported as CFU/ml. Data represent averages from three biological replicates, with three technical replicates per sample. Data were analyzed using ordinary one way ANOVA, using SIDAK’s multiple comparison test for comparisons between individual datasets. *P*-values were as follows: total adherent cells: 4 h *p* = 0.8640 (n.s.), 24 h ^∗∗∗∗^*p* =< 0.0001, invasion: 4 h ^∗∗∗^*p* = 0.0005, 24 h ^∗∗∗∗^*p* =< 0.0001.

In adhesion and invasion assays using the 16HBE14 bronchial epithelial cell line, total cell numbers (adherent and internalized cells, CFU/mL) were the same for HI2019^WT^ and HI2019^Δ^*^torZ^* after 4 h of incubation. However, after 24 h a statistically significant reduction in the CFU/mL for HI2019^Δ^*^torZ^* to ∼33% (*p* =< 0.0001, One-Way ANOVA) of the wild-type level was observed (**Figure 3**), indicating that HI2019^Δ^*^torZ^* has reduced ability to either colonize epithelial cells during longer incubation or to survive in contact with them. As the inoculum for these experiments came from cells grown on complete growth medium where HI2019^Δ^*^torZ^* shows no growth defect, it is possible that storage compounds may have contributed to growth of the mutant strain in the initial phases of the co-culture experiment. The reduction of adherence was also apparent in immunofluorescence stains of co-cultures (**Figure 3**). Invasion of 16HBE14 cells by HI2019^Δ^*^torZ^* (determined using a gentamicin protection assay) was also reduced after both 4 and 24 h incubation where 20% (*p* = 0.0005, One-Way ANOVA) and 29% (*p* =< 0.0001, One-Way ANOVA) of the respective wild-type CFU/mL were observed for HI2019^Δ^*^torZ^* (**Figure 3**). This suggests that the TorZ protein is either needed for intracellular survival or plays a role in the cell invasion process. Both of these functions are in keeping with a role in cellular energy generation via the respiratory chain.

**FIGURE 4:**
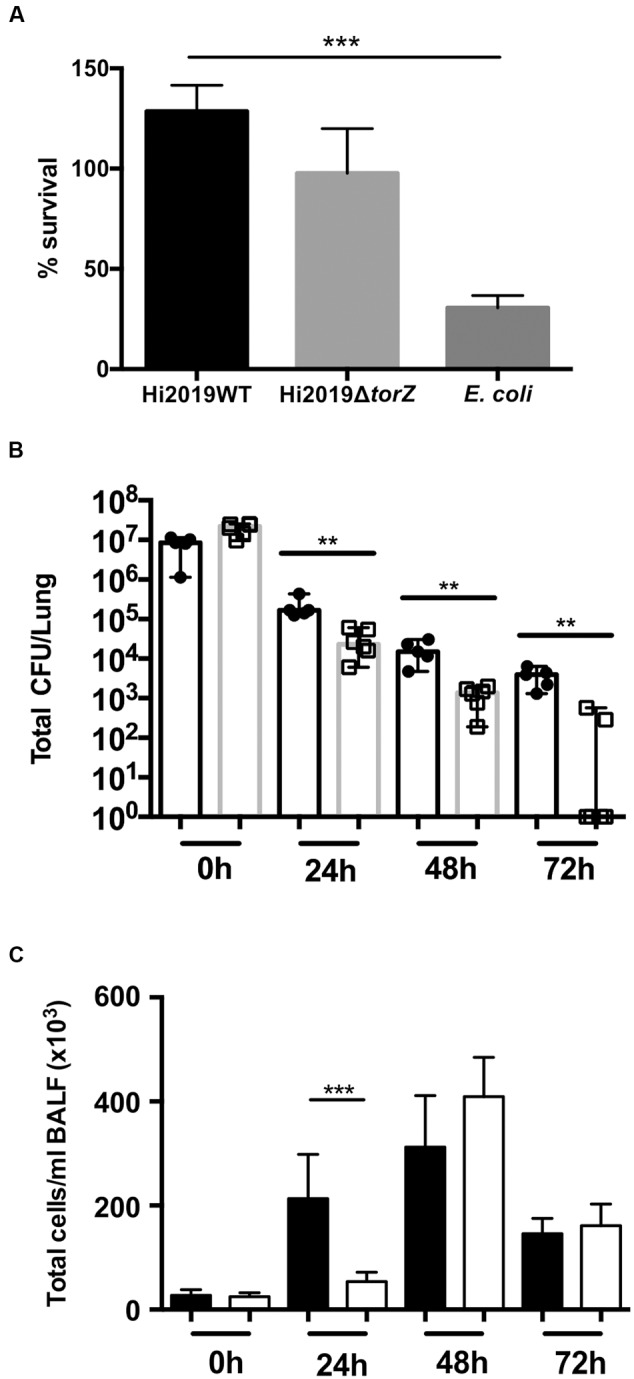
**Survival of HI2019^WT^ and HI2019^Δ^*^torZ^* in the respiratory tract of BALBc mice and in contact with primary human neutrophils. (A)** Survival in contact with primary human neutrophils following 2 h of incubation. *Escherichia coli* was used as a control. *P*-values determined by unpaired *t*-tests were HI2019^WT^ – *E. coli p* = 0.0003; HI2019^WT^ vs. HI2019^Δ^*^torZ^ p* = 0.1072 (n.s.). **(B)** Survival in the respiratory tract of BALBc mice. Mice were inoculated intranasally as *HI* is unable to cause a stable colonization of mice, a decrease of CFU/ml over time is expected. *P*-values determined by unpaired *t*-tests were 0 h ^∗∗^*p* = 0.00585, 24 h ^∗∗^*p* = 0.0083, 48 h ^∗∗^*p* = 0.00376, 72 h ^∗∗^*p* = 0.001865. **(C)** Immune cell counts in BALF fluid. Total cell counts are shown. Influx of immune cells is slower for HI2019^Δ^*^torZ^*, in keeping with the reduced fitness of this strain. ^∗∗∗^*p* = 0.0005.

Similar experiments were carried out using human primary neutrophils. After 2 h of incubation, HI2019^WT^ had increased to ∼128% of the originally used inoculum, while the level of HI2019^Δ^*^torZ^* was approximately the same (97%, *p* = 0.0949, One-Way ANOVA) as at the start of the experiment. *E. coli*, used as a control, decreased to 30% of the original inoculum in the 2 h incubation period (*p* = 0.0005 vs. HI2019^WT^) (**Figure 4**).

### Loss of TorZ Leads to Reduced HI Survival in a Murine Model of Infection

To determine if the *in vitro* survival defects were also observable *in vivo*, we then tested the ability of HI2019^Δ^*^torZ^* to survive in a murine model of bacterial clearance. Mice were infected intranasally with 10^7^ CFU and the number of remaining bacteria was assessed every 24 h up to 72 h relative to the wild type strain showing reduced survival of HI2019^Δ^*^torZ^* relative to the wild type strain (**Figure 4**). After 24 h incubation the cell numbers of the mutant strain were already reduced by 85% compared to the respective wild type levels at 24 h, and this reduction in CFU/mL for HI2019^Δ^*^torZ^* increased to 93 and 96% for the 48 and 72 h. In fact, at 72 h, four out of six mice infected with HI2019^Δ^*^torZ^* were culture negative, while 6/6 mice infected with HI2019^WT^ still reported on average 3.6 × 10^3^ CFU/mL (**Figure 4**). The HI2019^Δ^*^torZ^* strain also appeared to cause a slower immune response in the mice with significant influx of immune cells only observed after 48 h, compared to 24 h for HI2019^WT^ (**Figure 4**).

Taken together, our data indicate that the TorZ protein supports HI virulence, as a loss of the protein leads to reduced survival in a murine model of infection, and also reduced the ability of the bacteria to invade human cells. However, there is no information on the biological function of TorZ-like proteins in any bacterial species, and TMAO, the main substrate described for the *E. coli* TorZ (Gon et al., 2000), is not generally found in the respiratory tract. Therefore TMAO is unlikely to be the natural substrate for the HI enzyme, and a further characterization of HITorZ was required to determine whether the observed phenotypes were due to loss of oxidative damage repair through the TorZ substrate conversions or linked to the role of TorZ in the HI respiratory chain.

### *Haemophilus influenzae* TorZ Is a Periplasmic, S-isomer Specific Methionine Sulfoxide Reductase

The HI*torZ* gene encodes a twin arginine signal peptide, with a predicted cleavage site at position 41 (aa sequence: AVA_40_-_41_KE). This type of export signal targets the proteins for export via the membrane-bound TAT-system, all components of which are encoded in the HI2019 genome. Following cellular fractionation, ∼60% of the MetSO reductase activity was recovered in the periplasm of HI2019^WT^, while no significant activity was present in HI2019^Δ^*^torZ^* (Supplementary Figure S5). This clearly confirms that the observed activity was due to the presence of the functional, 87 kDaTorZ protein in the periplasm of *H. influenzae*.

**FIGURE 5:**
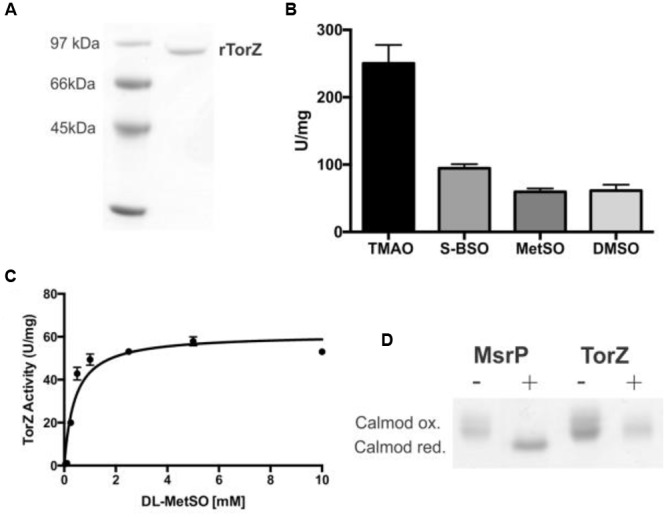
**Characterization of HI rTorZ. (A)** 10% SDS-PAGE of purified HI rTorZ; **(B)** activity of HI rTorZ with different N- and S-oxide substrates in standard assays. Averages shown are from at least three assays, errors are given as standard deviations. **(C)** Activity of HI rTorZ with increasing amounts of racemic DL-MetSO as substrate. Three repeat assays were carried out per concentrations shown, the data were fitted with Prism 6.0 (GraphPad) using direct non-linear fitting. Kinetic parameters are shown in **Table 2**. **(D)** Repair of oxidized calmodulin by HI rTorZ. *E. coli* MsrP was used as the positive control. Oxidized and reduced forms of Calmodulin have altered electrophoretic properties, the data show that HI rTorZ has no calmodulin reducing activity compared to MsrP. Labels: ‘+’ = complete assay containing reductant, enzyme and Calmodulin as the substrate, ‘-‘ = negative control without reductant to ensure the reaction is due to enzymatic activity.

The HI *torZ* gene without nucleotides 1–120 that encode the TorZ signal peptide was cloned into an expression plasmid, creating pProHITorZ-sp. Recombinant, soluble rTorZ protein was produced and purified with a yield of 5.6 mg of purified rTorZ from 4 L of expression culture (**Figure 5A**), and had a molybdenum content of 0.53 Mo atoms/TorZ molecule (or 0.53 moles Mo per mole TorZ) in the preparation as determined by ICP-OES. Based on gel filtration, the purified rTorZ was present as a monomer, and the optical absorbance spectra were similar to those of related Mo enzymes (Supplementary Figure S4) of the DMSO reductase family (Mcalpine et al., 1998), providing evidence that the Mo cofactor had been correctly inserted, and was able to interact with one of the proposed substrates, MetSO (Supplementary Figure S4, MetSO reduced spectrum).

Enzyme assays confirmed that rTorZ was able to reduce all four substrates tested previously, TMAO, DMSO, MetSO, and BSO (**Figure 5B**), showing that the enzyme is also an N-oxide reductase. We then tested the stereospecificity of rTorZ, as both MetSO and BSO exhibit diastereoisomerism due to the chirality of the S–O group that can be present in either an *R* or *S* form (Supplementary Figure S6). The substituted C-atom in the biotin bicyclic ring system alpha to the sulfoxide is always in a *S* configuration so only these two diastereoisomers (*RS* and *SS*) need to be considered. Racemic MetSO possesses four possible isomers due to chirality at the R-S(O)Me S-atom (*R* or *S*) and also the alpha C-atom of the amino acid (*S* or *R*) (Supplementary Figure S6). The *RR/SS* and *RS/SR* pairs are enantiomers.

rTorZ was able to reduce both racemic DL-MetSO (four isomers, Supplementary Figure S6) and L-MetSO (two isomers, both of the sulfoxide group) with similar rates, indicating that the enzyme is able to use both D- and L-MetSO as substrates. However, rTorZ reacted exclusively with *S*-BSO diastereoisomer (**Figure 5B**), while no activity with *R*-BSO was observed (data not shown). This suggests that the enzyme likely has a preference for S-sulfoxides over R-sulfoxides, and matches the observations made with crude extracts during the initial characterization of the HI2019^Δ^*^torZ^* strain (**Figure 2**). This stereospecificity can be rationalized on the basis of the structures of the two BSO isomers (Supplementary Figure S6). In the *RS*-BSO isomer the molecule adopts a distinct ‘U’-shaped conformation which creates a sterically congested environment for the O-atom of the sulfoxide that must ultimately coordinate to the Mo ion for catalytic reduction (de-oxygenation). Conversely, the extended conformation of SS-BSO creates a more accessible O-atom that can more easily coordinate to the active site of rTorZ.

Kinetic assays were used to clarify the natural substrate preference of rTorZ, and confirmed that the enzyme is a N- and S-oxide reductase, but is unlikely to be using the N-oxide TMAO as a natural substrate (**Table 2**; **Figure 5C**). While rTorZ had a high turnover rate with TMAO, this was only observed at physiologically irrelevant substrate concentrations (*K*_M_ > 6 mM). For the three S-oxides that were tested, turnover numbers differed significantly, between 183 s^-1^ for SS-BSO and ∼85–90 s^-1^ for DMSO and racemic-MetSO. The magnitude of the determined *K*_M_ values was inversely related to the magnitude of the *k*_cat_, with SS-BSO having a *K*_M_ of 1.2 mM, racemic MetSO of 0.41 mM for the diastereomeric mixture (0.205 mM corrected for S/R form of the sulfoxide) and DMSO of 0.14 mM. It should be noted here that for the racemic MetSO, both enantiomers of the sulfoxide group would have been present in the mixture and we were unable to resolve these. Given that rTorZ is only able to convert S-sulfoxides as shown by our experiments, the *K*_M_ reported here for racemic MetSO may be overestimating the actual *K*_M_, as only the *S*-MetSO molecules present in the assay would have been converted by rTorZ.

**Table 2:** Kinetic parameters of purified *HI* rTorZ for different S- and N-oxide substrates.

Substrate	*K*_M_ (mM)	*k*_cat_ (s^-1^)	*k*_cat_*/K*_M_ (s^-1^M^-1^)
TMAO	6.7 ± 2.0	434.7 ± 52.1	6.49 × 10^4^
DMSO	0.14 ± 0.05	85.4 ± 9.1	6.5 × 10^5^
DL-MetSO	0.41 ± 0.08	91.7 ± 4.4	2.24 × 10^5^
*S*-BSO	1.52 ± 0.28	182.6 ± 10.9	1.2 × 10^5^

*Errors are reported as standard deviations of the mean.*

These data indicate that rTorZ likely is a *S*-MetSO reductase, as, unlike DMSO, MetSO is a substrate that is present in the respiratory tract, and the affinity of rTorZ for MetSO is in a physiologically relevant range. While SS-BSO is another substrate that could occur in the human body, the *K*_M_ of well over 1 mM makes it unlikely that it is the natural substrate for this periplasmic enzyme. Conversion of MetSO is a novel function for molybdenum containing enzymes of the DMSO reductase family, and clearly differs from the described function of the *E. coli* TorZ as either a TMAO reductase or a biotin sulfoxide reductase.

### *Haemophilus influenzae* TorZ Appears to be Unable to Repair of MetSO–Damage to Proteins

Given its ability to reduce MetSO to Met, rTorZ might be involved in periplasmic protein repair following exposure to oxidative stress, and we tested this using an assay based on the Met-rich protein calmodulin and the MsrP MetSO reductase as a control (Gennaris et al., 2015). While MsrP was able to reduce calmodulin, under the same assay conditions, no reactivity of TorZ toward calmodulin was observed (**Figure 5D**). The assay used is very similar to the standard TorZ kinetic enzyme assay, except that calmodulin was added as the substrate, and thus should have been suitable for TorZ reactivity. While we cannot completely rule out that TorZ might be able to react with other oxidized proteins, this result strongly suggests that protein repair is not the primary enzymatic function of TorZ, and instead the observed phenotypes may be related to the function of TorZ in the HI respiratory chain.

### Phylogenetic Analyses of *H. influenzae* rTorZ Confirm That it Is a Novel Type of Mo-containing S-oxide Reductase

The data collated above clearly highlight that HITorZ differs from other Mo enzymes in both the preferred catalyzed reaction and its effects on bacterial physiology. To determine whether rTorZ is a novel type of Mo enzyme or a variant of a known type, we analyzed the phylogenetic relationship of HITorZ to other characterized enzymes of the Dor/Tor type described in the literature (Supplementary Figure S7). This showed that HITorZ shares a common ancestor with *E. coli* BisC (Pierson and Campbell, 1990; Barber et al., 1992) and TorZ (Gon et al., 2000), but resides on a separate branch within that group, and does not group with the *E. coli* TorZ sequences (Supplementary Figure S7). This is also reflected in the sequence identities that were about 40–50% for all enzymes in the alignment relative to HITorZ (53% EcTorZ; 49% EcBisC; 42% EcTorA, and 48% RcDorA), which is a moderate degree of conservation, reflecting the common phylogenetic origin of enzymes within this group. In keeping with previous published work, all S-oxide reductases in the alignment contained residues equivalent to Tyr114 and Trp116 (Rc DorA numbering), which were identified as crucial active site residues in *R. capsulatus* DorA (Mcewan et al., 2003). In the *E. coli* N-oxide reductase TorA Tyr114 is absent. The aspartate ligand to the Mo cofactor (Mcalpine et al., 1997) was also conserved in all sequences.

We then expanded the alignment to include a total of 1005 sequences of proteins of the Dor/Tor group of enzymes (see Supplementary Data, **Figure 6**) to obtain a global view of enzyme evolution within this group, and were able to identify three major phylogenetic groups of enzymes within the Dor/Tor group of Mo enzymes. Group I contained sequences related to *E. coli* TorA (Group 1A, also included sequences from *Serratia sp.; Photobacterium* sp.; *Aeromonas* sp.; *Citrobacter* sp., *Pasteurella multocida*, and *Shewanella* sp.) and *R. capsulatus* DorA (Group 1B, *Burkholderia* sp.; *Rhodopseudomonas* sp., *Cupriavidus* sp., and *Rhodobacter* sp.) respectively, while group III had five subgroups: (i) Group 3A: *E. coli* BisC sequences and homologs from *S. enterica* and *Citrobacter* sp.; (ii) Group 3B *Klebsiella* sp. and *Enterobacter* sp. sequences; (iii) Group 3C *Cronobacter* sp. sequences; (iv) Group 3D *Vibrio* sp. and *Citrobacter* sequences; and (v) Group 3E *E. coli* TorZ sequences. Whether the proteins in groups 3B–3D encode enzymes with *E. coli* BisC- or TorZ-like activities remains to be analyzed, however, as *E. coli* TorZ was originally described as a biotin sulfoxide reductase, we have tentatively called this group the biotin sulfoxide reductase group of sequences.

**FIGURE 6:**
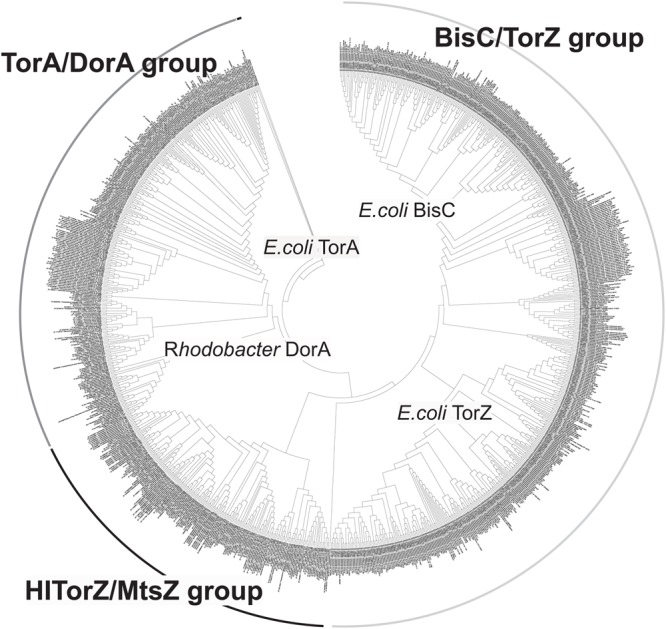
**Phylogenetic analysis of HITorZ/MtsZ related protein sequences of the DMSO reductase enzyme family.** HITorZ/MtsZ clearly forms a distinct group within this subgroup of DMSO reductase enzyme family enzymes as indicated by the branching pattern. The phylogenetic tree shown was constructed from a total of 1005 sequences retrieved from NCBI by BLAST searches (see Supplementary Data) using the neighbor-joining algorithm. The phylogenetic analyses used MEGA 6.0, bootstrapping used 500 replicates.

Group II (putative MetSO reductases) contained the sequences related to the HI TorZ protein which formed two large subgroups. Group 2A contained sequences from *Yersinia* sp., *Serratia* sp., *Enterobacter* sp., *Photobacterium* and *Aeromonas* sp., while Group 2B was dominated by sequences originating from various species of Pasteurellaceae, including HI, and in addition contained some sequences from *Campylobacter* sp. and *Helicobacter* sp.. These data clearly indicate that HITorZ protein is a unique protein within the Dor/Tor-type enzymes of the DMSO reductase family, and based on its apparent preference for MetSO as a substrate we propose that the HITorY and TorZ proteins be renamed to MtsY and MtsZ (Mts, **M**e**t**hionine**s**ulfoxide) to clearly distinguish them from the *E. coli* TorY and TorZ.

## Discussion

The DMSO reductase family of molybdenum enzymes contains a number of enzymes that are able to reduce N- and S-oxides, and in bacteria that can live freely or in association with a host such as *E. coli*, TMAO and DMSO are usually considered as the physiological substrates for these enzymes since the former is a nitrogen waste product found in a variety of animals, especially fish, while the latter is a component of the organo-sulfur cycle (Kappler and Schaefer, 2014). However, for an obligate host-adapted bacterium such as HI the nature of the physiological substrate for S-/N-oxide reductases is less obvious since this bacterium is unlikely to encounter DMSO and TMAO in its natural environment.

The HI MtsZ protein that we have characterized here is a novel, previously uncharacterized molybdenum enzyme of the DMSO reductase family that uses MetSO as its main substrate. Unlike the periplasmic DMSO and TMAO reductases (Iobbi-Nivol et al., 1996; Gon et al., 2000; Mcewan et al., 2004) to which it is related, MtsZ is prevalent in pathogenic bacteria, especially of the Pasteurellaceae family of bacterial pathogens, where proteins related to HIMtsZ are found in every major group of this family (**Figure 6**).

Catalytically, purified MtsZ protein showed a clear preference for S-oxides rather than N-oxides based on measurements of *k*_cat_/*K*_M_ in combination with assessment of *K*_M_ values (**Table 2**), with DMSO and MetSO being the only substrates tested with *K*_M_ values in a physiologically relevant concentration range which is essential for cellular control of enzyme activity. MtsZ reacted specifically with sulfoxides in S-conformation, and thus has the same stereospecificity as the related *R. capsulatus* DorA DMSO reductase (Hanlon et al., 1998) and the BisC biotin sulfoxide reductases from *E. coli* and *Salmonella* (Denkel et al., 2013).

Interestingly, MtsZ appears to be specific for small molecule S-oxides such as free MetSO and DMSO rather than oxidized Met residues that form in many periplasmic proteins during oxidative stress and thus has a clearly different cellular function from the only other known Mo-containing MetSO reductase, MsrP, that was recently described and appears to be specific for repair of MetSo residues in periplasmic protein repair. MsrP was characterized in *E. coli*, and belongs to an unrelated group of Mo-containing enzymes (Gennaris et al., 2015).

**FIGURE 7:**
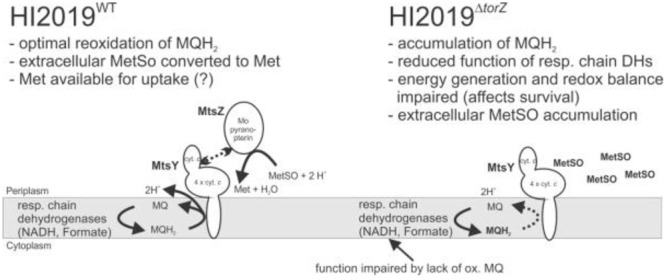
**Current model of HIMtsZ physiological function in HI2019^WT^ and HI2019^Δ^*^torZ^*.** MetSO, methionine sulfoxide; Met, methionine; MQ, oxidized menaquinone; MQH_2_, reduced menaquinone. MQH_2_ in bold (right panel) indicates accumulation of this compound due to lack of MtsZ activity.

Based on what we know so far, there are two possible functions for HIMtsZ. First, the enzyme might support HI oxidative stress defenses by re-reducing oxidized S-oxides, e.g., by converting MetSO back to Met, which is the form in which it can be used by cells. Secondly, the MtsZ catalyzed reaction is connected to the respiratory chain via the pentaheme cytochrome MtsY and thus can also support respiration and redox balancing in HI under oxygen-limited conditions, as indicated by the enzyme activities and gene expression profiles (**Figures 1** and **7**).

In support of the first point, free methionine, the precursor of the natural substrate of MtsZ has been detected in human serum (Kermack et al., 2015). The sulfur group in methionine is easily oxidized and this oxidation can be associated with ROS or the action of enzymes such as myeloperoxidase (MPO) which is released by human neutrophils and catalyzes the production of hypochlorite (HOCl) from peroxide and chloride ions (Drozdz et al., 1988). While epithelial cells are not major producers of ROS it has been shown that they are able to produce ROS especially when stimulated by LPS, and that this may involve NOX or DUOX-type NADPH Oxidases (Boncompain et al., 2010; Uy et al., 2011; Rada et al., 2014) and our gene expression data from co-cultures clearly show high levels of *mtsZ* transcription in co-cultures. Converting molecules such as MetSO back to their reduced state could contribute to the formation of an extracellular oxidative stress buffer, as Met will react quickly with ROS or HOCl.

However, our data also show that MtsZ does not appear to be involved in repair of MetSO damage to proteins as indicated by a lack of increased HOCl sensitivity and inability to repair oxidized calmodulin, showing that the HI2019^MtsZ^ mutant strain still contained a fully functional system for repair of HOCl induced oxidative damage.

Despite this, the MtsZ mutant strain showed clear defects in a number of assays, such as biofilm formation and survival, adherence and invasion to host cells and survival in a murine model of infection. The pivotal role of MtsZ in survival of HI2019 in co-cultures with host cells and a mouse model suggests that these interactions might take place under oxygen-limitation, when *mtsZ* expression is high. In the co-culture experiments, the reduced ability of HI2019^Δ^*^torZ^* to invade the human tissue cells could either be a follow on effect from a reduced number of adherent cells, reflect a loss of the ability of the strain to be taken up into the cells or a loss of the ability to survive intracellularly. All of these phenotypes are consistent with a role for the MetSO reductase activity of MtsZ in support of HI energy generation and redox balancing during growth under limiting oxygen conditions, including during contact with host cells.

A similar observation has been made for the BisC biotin sulfoxide reductase from *S. enteria* serovar Typhimurium, where BisC activity was required for repair of biotin sulfoxide and, to a lesser extent, MetSO during growth inside macrophages (Denkel et al., 2013). The *Salmonella* Δ*bisC* strain had attenuated survival in a mouse model, with a stronger attenuation being observed in mice that had a more potent oxidative response (Denkel et al., 2013).

If free MetSO is the substrate of MtsZ, then MtsZ might also have a role in ensuring cellular methionine supplies. A possible side effect of oxidative stress can be an inactivation of the methionine biosynthesis through damage to the MetE protein, as has been observed in *E. coli* (Hondorp and Matthews, 2004; Leichert and Jakob, 2004). This inactivation of MetE renders the bacteria temporarily unable to synthesize methionine *de novo*, and in such a scenario MtsZ could also be involved in enabling scavenging of functional methionine from the extracellular environment. Interestingly, methionine biosynthesis genes including *metE*, have been shown to support virulence in another respiratory pathogen, *Streptococcus pneumoniae* (Basavanna et al., 2013).

In summary, our results clearly show that MtsZ is a previously uncharacterized type of molybdenum enzymes with a high degree of conservation in the Pasteurellaceae family of bacterial pathogens, and that the MtsZ protein and/or the associated enzymatic activities (**Figure 2**) are required for HI interactions with host cells both *in vitro* and *in vivo*. Given the striking effect of a loss of MtsZ on survival of HI in contact with host cells and its periplasmic location this protein might be a useful target of inhibitors for the prevention of HI infection. Our current model for MtsZ function is that this enzyme is required for optimal anaerobic energy generation and maintenance of a redox balance in HI, and this in turn compromises HI survival in complex environments such as biofilms, in contact with host cells or animal model tissues (**Figure 7**). By recovering oxidized methionine in the periplasm of the HI cells, MtsZ may also contribute to either scavenging of functional Met, or creation of a ‘redox buffer’ for prevention of damage to other cellular components in the HI extracellular space. Despite possessing a suitable catalytic activity for repair of proteins with oxidative damage, this does not appear to be a main role for MtsZ.

Future work on this system should include investigations of possible changes in host cell responses to exposure to an HI strain lacking TorZ, as well as further work on the mechanism of action of MtsZ, to fully understand how it supports virulence of HI.

## Author Contributions

UK coordinated the research, supervised all students and staff involved, UK, RD, and AM prepared the main manuscript drafts edited figures and contributed to data analyses. RD carried out the majority of experiments on co-cultures and enzyme characterization. DKSMPO carried out the initial analyses of torZ expression and generation of the knockout mutant strains. AD, VL, MN, HW, and XL carried out the phylogenetic analyses, preparation of TorZ expression plasmids and optimized protein expression and purification. PB prepared purified sulfoxide substrates for enzyme assays. A-TE and PH carried out the mouse experiments an associated data analyses. All authors reviewed the manuscript and contributed to the drafting of sections relevant to their work.

## Conflict of Interest Statement

The authors declare that the research was conducted in the absence of any commercial or financial relationships that could be construed as a potential conflict of interest.

## Footnotes

1 www.doubling-time.com, (accessed 23/07/2016)
