# Supplementary material for: A Novel, Molybdenum-Containing Methionine Sulfoxide Reductase Supports Survival of Haemophilus influenzae in an In vivo Model of Infection
Source: Front Microbiol. 2016 Nov 14;7:1743. doi: 10.3389/fmicb.2016.01743 (PMC5122715; doi:10.3389/fmicb.2016.01743)
Supplement: Supplementary file 1 [file Table_1.docx]

| *H. influenzae* strain | genome complete | *torZ* | gene locus *torZ* | *torY* | gene locus *torY* |
| --- | --- | --- | --- | --- | --- |
| RDKW20 | ✓ | ✓ | HI0643 | ✓ | HI0644 |
| PittEE | ✓ | ✓ | CGSHIEE_RS08530 | ✓ | CGSHIEE_RS08525 |
| R2866 | ✓ | ✓ | R2866_RS09220 | ✓ | R2866_RS09215 |
| R2846 | ✓ | ✓ | R2846_RS08595 | ✓ | R2846_RS08590 |
| F3047 | ✓ | ✓ | HICON_RS02605 | ✓ | HICON_RS02600 |
| KR494 | ✓ | ✓ | HIFGL_RS01380 | ✓ | HIFGL_RS01385 |
| CGSHiCZ412602 | ✓ | ✓ | H733_RS02780 | ✓ | H733_RS02785 |
| Hi375 | ✓ | ✓ | NF38_RS01915 | ✓ | NF38_RS01910 |
| 477 | ✓ | ✓ | NTHI477_RS08650 | ✓ | NTHI477_RS08655 |
| C486 | ✓ | ✓ | NTHIC486_RS01765 | ✓ | NTHIC486_RS01760 |
| 2019 | ✓ | ✓ | AAY75_RS05150 | ✓ | AAY75_RS05155 |
| PittGG | ✓ | ✓ | pseudogene | ✓ | CGSHiGG_06495 |
| 86-028NP | ✓ |  |  |  |  |
| F3031 | ✓ |  |  |  |  |
| 10810 | ✓ |  |  |  |  |
| 723 | ✓ |  |  |  |  |
| Hi361 |  | Pgene | SU52_RS07445 | ✓ | SU52_RS07450 |
| MiHi270 |  | Pgene | SU57_RS00095 | ✓ | SU57_RS00100 |
| Hi381 |  | Pgene | SU54_RS03075 | ✓ | SU54_RS03080 |
| 7P49H1 |  | Pgene | CGSHI7P49H1_RS008880 | ✓ | CGSHI7P49H1_RS00555 |
| 3655 |  | ✓ | CGSHI3655_RS00510 | Pgene | CGSHI3655_RS00505 |
| PittAA |  | ✓ | CGSHIAA_RS01560 | ✓ | CGSHIAA_RS01555 |
| PittHH |  | ✓ | CGSHIHH_RS07850 | ✓ | CGSHIHH_RS07855 |
| PittII |  | ✓ | CGSHIII_RS03355 | ✓ | CGSHIII_RS03350 |
| NT127 |  | ✓ | HIAG_RS01085 | ✓ | HIAG_RS01080 |
| RdAW |  | ✓ | HICG_RS00900 | ✓ | HICG_RS00905 |
| 584 |  | ✓ | CN10_RS00360 | ✓ | CN10_RS00355 |
| 411 |  | ✓ | CK45_RS05915 | ✓ | CK45_RS05920 |
| 1104 |  | ✓ | CN11_RS03245 | ✓ | CN11_RS03250 |
| 60294N1 |  | ✓ | W820_RS02390 | ✓ | W820_RS02395 |
| RMHi93 |  | ✓ | SU59_RS07425 | ✓ | SU59_RS07430 |
| MiHi64 |  | ✓ | SU58_RS05175 | ✓ | SU58_RS05180 |
| Hi322 |  | ✓ | SU30_RS09335 | ✓ | SU30_RS09330 |
| Hi359 |  | ✓ | SU51_RS04220 | ✓ | SU51_RS04225 |
| Hi345 |  | ✓ | SU50_RS01695 | ✓ | SU50_RS01700 |
| Hi378 |  | ✓ | SU53_RS00295 | ✓ | SU53_RS00290 |
| Hi394 |  | ✓ | SU55_RS08675 | ✓ | SU55_RS08680 |
| Hi403 |  | ✓ | SU56_RS08615 | ✓ | SU56_RS08620 |
| 1059_HINF |  | ✓ | AC948_RS05830 | ✓ | AC948_RS05835 |
| 1057_HINF |  | ✓ | AC980_RS06820 | ✓ | AC980_RS06815 |
| 1061_HINF |  | ✓ | AC985_RS00835 | ✓ | AC985_RS00840 |
| 1124_HINF |  | ✓ | AC990_RS07120 | ✓ | AC990_RS07125 |
| 536_HINF |  | ✓ | ADA60_RS09515 | ✓ | ADA60_RS09520 |
| 615_HINF |  | ✓ | ADA64_RS05930 | ✓ | ADA64_RS05935 |
| 177_HINF |  | ✓ | ADA86_RS06235 | ✓ | ADA86_RS06230 |
| 552_HINF |  | ✓ | ADB36_RS07875 | ✓ | ADB36_RS07880 |
| 1123_HINF |  | ✓ | ADM46_RS05810 | ✓ | ADM46_RS05815 |
| HI1988 |  | ✓ | ABN83_RS05360 | ✓ | ABN83_RS05365 |
| HI2004 |  | ✓ | ABN75_RS01970 | ✓ | ABN75_RS01975 |
| HI2007 |  | ✓ | ABN76_RS08485 | ✓ | ABN76_RS08490 |
| HI2116 |  | ✓ | ABN66_RS08835 | ✓ | ABN66_RS08840 |
| HI1722 |  | ✓ | ABN58_RS05585 | ✓ | ABN58_RS05590 |
| HI1980 |  | ✓ | ABN79_RS03695 | ✓ | ABN79_RS0369 |
| HI1426 |  | ✓ | ABN54_RS08455 | ✓ | ABN54_RS08450 |
| HI1417 |  | ✓ | ABN52_RS05400 | ✓ | ABN52_RS05395 |
| HI1394 |  | ✓ | ABN40_RS00295 | ✓ | ABN40_RS00300 |
| HI1388 |  | ✓ | ABN35_RS02900 | ✓ | ABN35_RS02905 |
| HI1373 |  | ✓ | ABN27_RS00485 | ✓ | ABN27_RS00490 |
| HI1408 |  | ✓ | ABN48_RS09215 | ✓ | ABN48_RS09220 |
| C10 |  | ✓ | ABW52_RS05410 | ✓ | ABW52_RS05405 |
| HI1413 |  | ✓ | AC246_RS04425 | ✓ | AC246_RS04430 |
| HI2428 |  | ✓ | AC250_RS08240 | ✓ | AC250_RS08245 |
| HI2192 |  | ✓ | AC248_RS08170 | ✓ | AC248_RS08165 |
| 22.1-21 |  | ✓ | CGSHi22121_01502 | ✓ | CGSHi22121_01487 |
| R3021 |  | ✓ | CGSHi22421_05072 | ✓ | CGSHi22421_05077 |
| 6P18H1 |  |  |  |  |  |
| 137_HINF |  |  |  |  |  |
| 156_HINF |  |  |  |  |  |
| 159_HINF |  |  |  |  |  |
| 40_HINF |  |  |  |  |  |
| 841_HINF |  |  |  |  |  |
| 781_HINF |  |  |  |  |  |
| 839_HINF |  |  |  |  |  |
| 167_HINF |  |  |  |  |  |
| 492_HINF |  |  |  |  |  |
| HI2114 |  |  |  |  |  |
| HI1974 |  |  |  |  |  |
| HI1374 |  |  |  |  |  |
| HK1212 |  |  |  |  |  |
| 22.4-21 |  |  |  |  |  |
