# Supplementary material for: A Novel, Molybdenum-Containing Methionine Sulfoxide Reductase Supports Survival of Haemophilus influenzae in an In vivo Model of Infection
Source: Front Microbiol. 2016 Nov 14;7:1743. doi: 10.3389/fmicb.2016.01743 (PMC5122715; doi:10.3389/fmicb.2016.01743)
Supplement: Supplementary file 3 [file Image_1.pdf]

A novel, molybdenum-containing methionine sulfoxide reductase supports survival of *Haemophilus influenzae* in an *in vivo* model of infection

Rabeb Dhouib[1], D.K Seti Maimonah Pg Othman[1], Victor Lin[1], Xuanjie Jason Lai[1], Hewa Godage Sithija Wijesinghe[1], Ama-Tawiah Essilfie[2], Amanda Davis[1,3], Marufa Nasreen[1], Paul V. Bernhardt[1], Philip M. Hansbro[2], Alastair G. McEwan[1], Ulrike Kappler[1*]

[1] School of Chemistry and Molecular Biosciences, Centre for Metals in Biology, Australian Infectious Diseases Research Centre, The University of Queensland, St. Lucia, QLD 4072, Australia.
[2] Centre for Asthma and Respiratory Diseases and Hunter Medical Research Institute, The University of Newcastle, Locked Bag 1000, New Lambton, NSW 2305, Australia.
[3]Department of Chemistry and Biochemistry, University of Arizona, 1306 E. University Blvd., Tucson, Arizona 85721-0041, U.S.A.

Supplementary Figures

Figure S1: Growth rates of HI2019^{WT} and HI2019$^{\Delta torZ}$ in CDM glucose under aerobic, microaerophilic and anaerobic conditions as described in Othman et al. Front Microbiology 2014, doi:10.3389/fmicb.2014.00069. Bar represent averages from growth of 3 biological replicates, error bars: standard deviation.

Figure S2: In vitro biofilm formation by HI2019^{WT}, HI2019$^{\Delta torZ}$ and HI2019$^{\Delta torZ_comp}$. Both biofilm density (left hand side, monitored by crystal violet staining) and CFU present in the biofilms (right hand side) were monitored. The analyses used 96 well plates and three different aeration regimes. Statistical significance analyses used one-way ANOVA with a p= 0.05 cut off or statistical significance. Error bars represent the STDEV of the mean, p-values are **** = <0.0001; ***= < 0.001, **=<0.01, * =< 0.05.

Figure S3: Expression of *torZ* in Hi2019WT during co-culture with 16HBE14 cells. RNA was isolated from planktonic and adherent bacteria. As controls, expression values for *rpoD* (housekeeping sigma factor) and *zwf* (glucose degradation via Pentose Phosphate Pathway) are shown. Neither of these genes shows a strong variation in expression levels, while *torZ* showed significantly higher expression than *zwf*, a central metabolic gene, and was more highly expressed in the planktonic bacteria. Expression data were normalized against *gyrA* gene expression.

Figure S4: Optical spectra of purified recombinant TorZ as prepared (ap) and in the presence of DL-MetSO.

Figure S5: Methionine Sulfoxide Reductase (MetSOR) activity in periplasmic and cytoplasmic fractions of HI2019^{WT} and HI2019$^{\Delta torZ}$. Enzyme activities are reported as U/mg, each determination was repeated three times, errors are given as standard deviations of the mean.

Figure S6: Structures and chirality of biotin- and methionine sulfoxides

Figure S7: Phylogenetic analysis of HiTorZ including only characterized N- and S-oxide reductase sequences. Algorithm: Neighbor joining, Bootstrap – 500 replicates. Sequence accession numbers: *Rcaps* DorA AAD13674., *E. coli* TorA AAC74082.1, *E. coli* TorZ AAC74942.2, *E. coli* BisC AAC76575.3, *E. coli* DmsA P18775.2, HI2019 DmsA WP_046067716.1, HI2019 TorZ AKA46614.1.

Figure S1

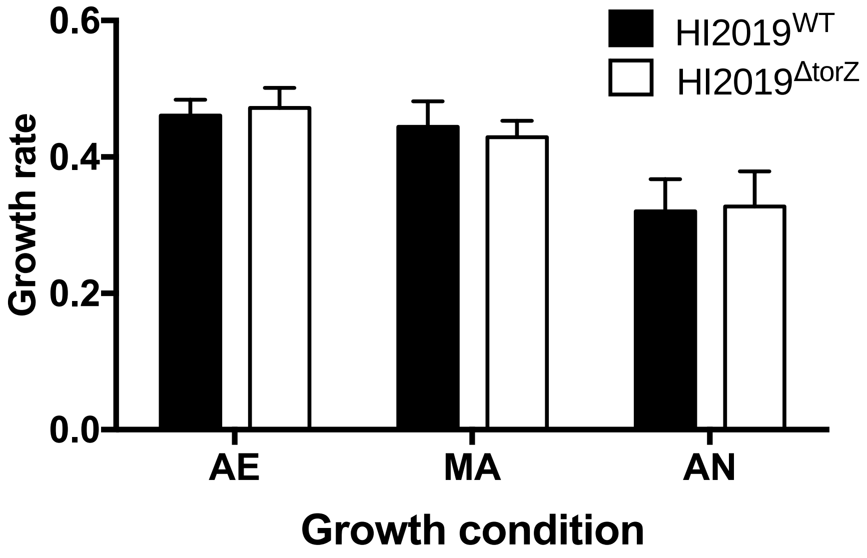

HI2019WT
HI2019ΔtorZ
0.6
0.4
0.2
0.0
Growth rate
AE
MA
AN
Growth condition

Figure S2

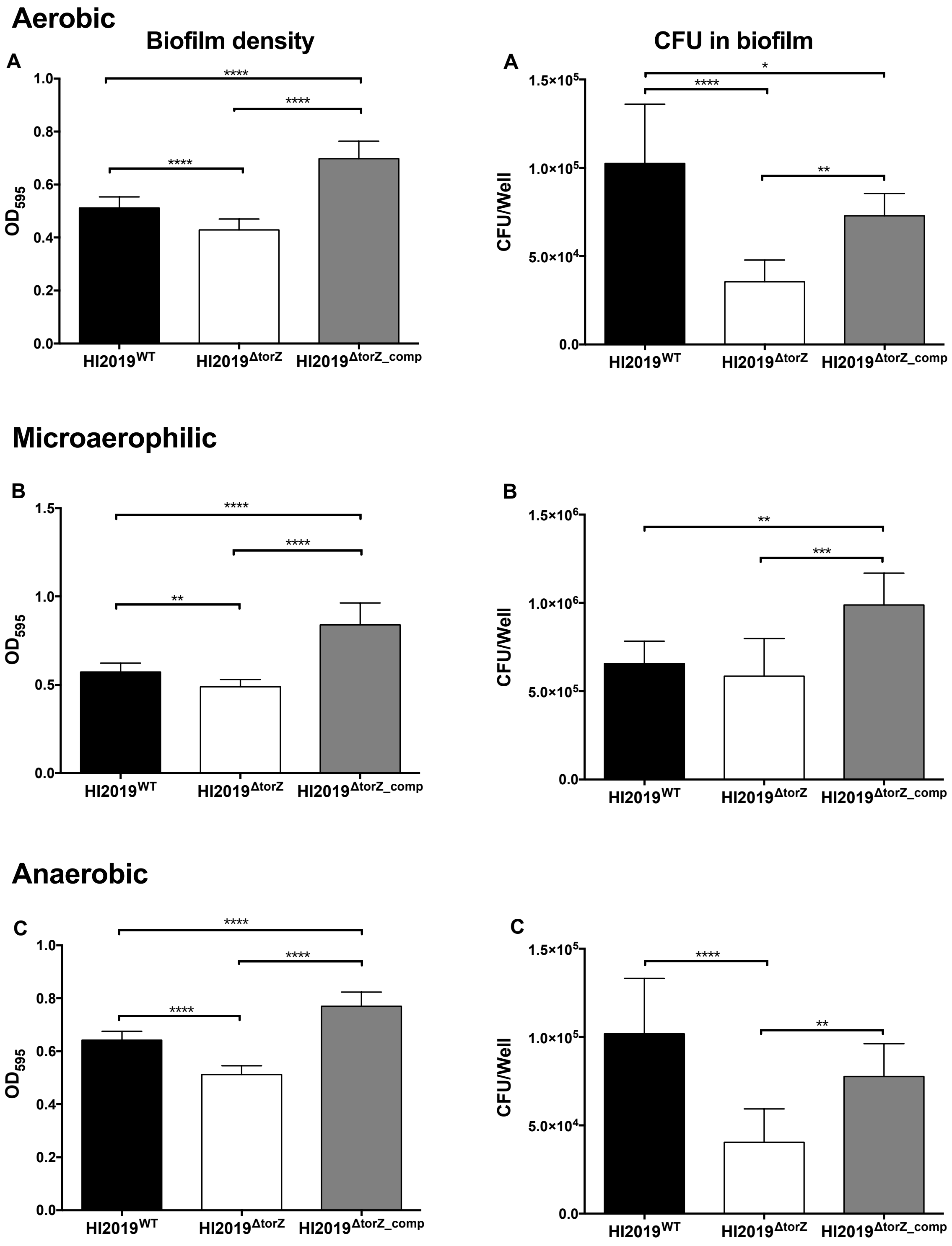
Aerobic
Biofilm density
CFU in biofilm
A
A
1.0
0.8
0.6
0.4
0.2
0.0
OD595
****
****
****
HI2019WT
HI2019ΔtorZ
HI2019ΔtorZ_comp
1.5×10⁵
1.0×10⁵
5.0×10⁴
0.0
CFU/Well
*
****
**
Microaerophilic
B
B
1.5
1.0
0.5
0.0
**
1.5×10⁶
1.0×10⁶
5.0×10⁵
***
Anaerobic
C
C

Figure S3

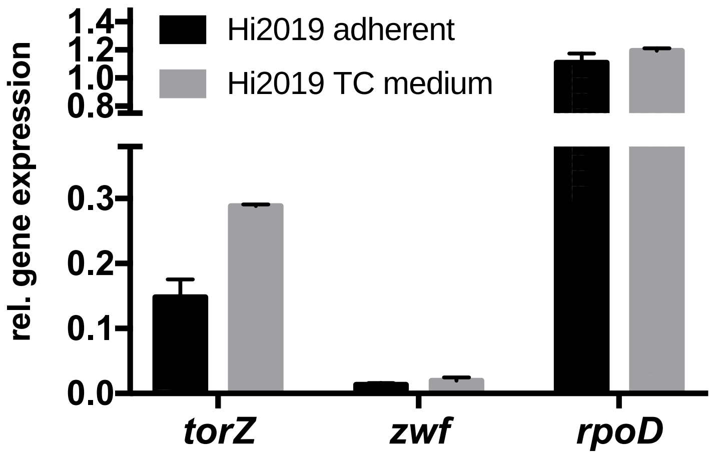
Hi2019 adherent
Hi2019 TC medium
rel. gene expression
1.4
1.2
1.0
0.8
0.3
0.2
0.1
0.0
torZ
zwf
rpoD

Figure S4

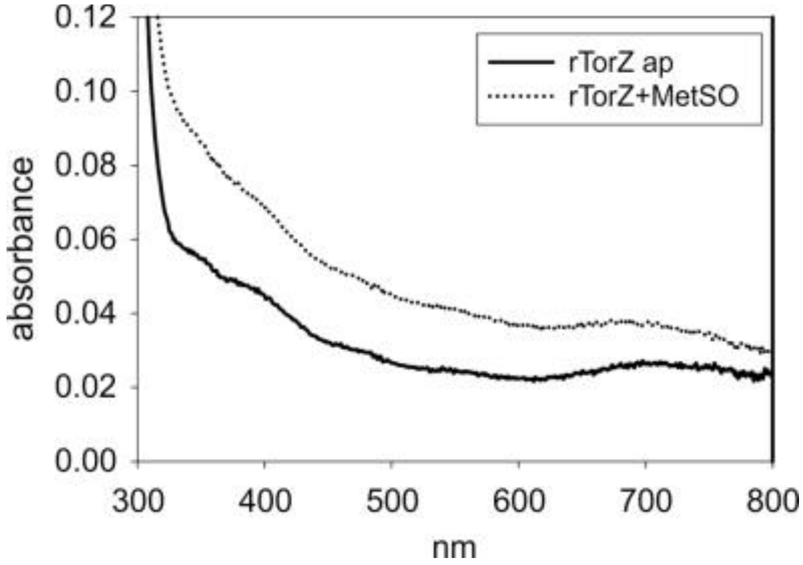
rTorZ ap
rTorZ+MetSO
absorbance
0.12
0.10
0.08
0.06
0.04
0.02
0.00
300
400
500
600
700
800
nm

Figure S5

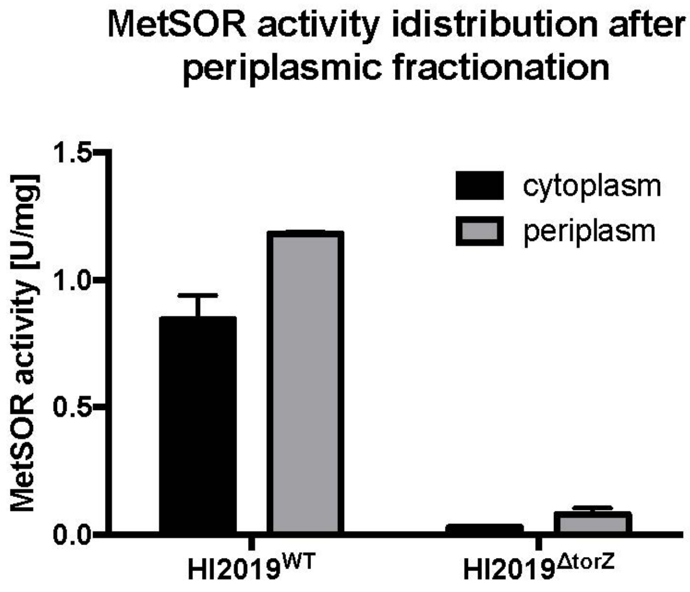

MetSOR activity idistribution after
periplasmic fractionation
MetSOR activity [U/mg]
1.5
1.0
0.5
0.0
cytoplasm
periplasm
HI2019WT
HI2019ΔtorZ

Figure S6

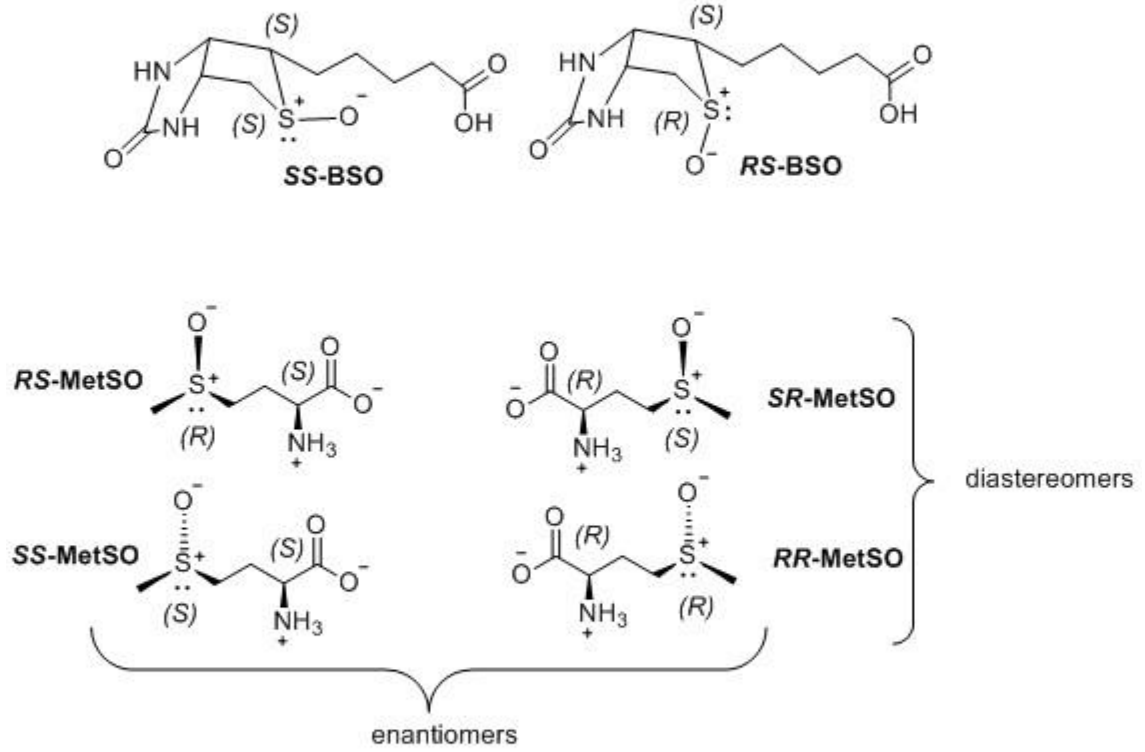

(S)
HN
NH
O
(S)
O
OH
SS-BSO
(S)
HN
NH
O
(R)
O
OH
RS-BSO
RS-MetSO
(R)
(S)
NH3
SR-MetSO
(R)
NH3
(S)
diastereomers
SS-MetSO
(S)
(S)
NH3
(R)
NH3
(R)
RR-MetSO
enantiomers

Figure S7

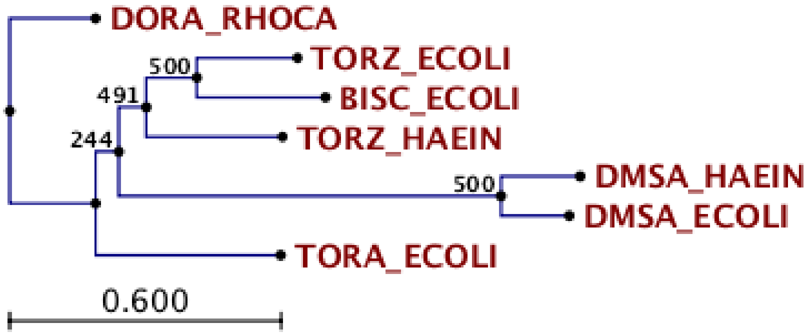
DORA_RHOCA
TORZ_ECOLI
BISC_ECOLI
TORZ_HAEIN
DMSA_HAEIN
DMSA_ECOLI
TORA_ECOLI
500
491
244
500
0.600
